# Nanospikes-mediated Anomalous Dispersities of Hydropobic Micro-objects and their Application for Oil Emulsion Cleaning

**DOI:** 10.1038/s41598-018-30339-3

**Published:** 2018-08-22

**Authors:** Hui-Jiuan Chen, Chengduan Yang, Tian Hang, Guishi Liu, Jiangming Wu, Di-an Lin, Aihua Zhang, Yan Li, Bo-ru Yang, Xi Xie

**Affiliations:** 1 The First Affiliated Hospital of Sun Yat-Sen University, State Key Laboratory of Optoelectronic Materials and Technologies, School of Electronics and Information Technology, Guangdong Province Key Laboratory of Display Material and Technology, Sun Yat-Sen University, Guangzhou, China; 2 Department of Dermatology, Massachusetts General Hospital, Harvard Medical School, Boston, MA USA

## Abstract

Many fields of applications require dispersion of hydrophobic particles in water, which is traditionally achieved by using surfactants or amphiphilic molecules to modify particle surfaces. However, surfactants or amphiphilic molecules may disturb the native solution or particles’ surface hydrophobicity, limiting extended applications such as oil emulsion cleaning. Recently one example of 2 μm-size polystyrene microparticles covered with ZnO nanospikes has been shown to exhibit excellent dispersity in water in spite of surface hydrophobicity. Whether this anomalous dispersion phenomenon was applicable to other hydrophobic microparticle systems was still unclear and its application scope was limited. Here the anomalous dispersities of different hydrophobic spiky micro-objects were systematically explored. The results show that the anomalous dispersion phenomenon was universally observed on different hydrophobic spiky micro-objects including different hydrophobic coating, particle sizes, material compositions and core particle morphologies. In addition, the spiky micro-objects displayed anomalous dispersity in water without compromising surface hydrophobicity, and their applications for oil spills absorption and oil emulsion cleaning were demonstrated. This work offers unique insight on the nanospikes-mediated anomalous dispersion phenomenon of hydrophobic micro-object and potentially extends its applicability and application scopes.

## Introduction

Many fields of applications such as oil spills/emulsion cleaning^1–3^, catalysts^4,5^, and hydrophobic surface coating^6–9^ *et al*. require dispersion of hydrophobic particles in aqueous solution. Due to surface hydrophobic attractions and van der Waals force, hydrophobic particles tend to aggregate in aqueous solution rather than presenting dispersion profiles. The particle aggregations were generally difficult to re-suspend even with strong-power sonication. Traditionally dispersion of hydrophobic particles in aqueous solution was achieved by using surfactants^10–12^ or amphiphilic molecules^13–15^ to modify particle surfaces to be hydrophilic. However, the using of surfactants or amphiphilic molecules has been suffering from several issues^16–19^. Surfactants as additives generally require a large amount to supplement into the native solution to reach a critical concentration for camouflaging particle surface, and this may induce undesirable impacts to the native solution. In addition, the application conditions of surfactants or amphiphilic molecules are highly dependent on the particular particle and the solution systems, highly varying when extended to other systems. On the other hand, if the particle surface was modified with amphiphilic molecules for dispersion purpose, the modification essentially altered particle’s surface hydrophobicity to be hydrophilic, and this disrupted the surface hydrophobicity for extended applications. For example, in spite of the success of oil spills absorption with hydrophobic micromaterials, *in-situ* cleaning of oil emulsion suspended in water remained challenging. When hydrophobic microparticles were applied as absorbers for cleaning of oil emulsion, the hydrophobic microparticles tended to aggregate in water with limited exposed hydrophobic surface for oil absorption^20–23^. If the hydrophobic microparticles were conjugated with surfactants for enhanced dispersion in water, the surfactant coating might reduce the surface hydrophobicity and compromise the oil absorption capability.

Recently, polystyrene (PS) microparticles attached with ZnO nanospikes, namely “hedgehog particles”, were found to exhibit anomalous dispersity, where excellent dispersity of hydrophobic microparticles in water was achieved without using surfactants^24^. This anomalous phenomenon was hypothesized to attribute to the nanospikes providing steric hindrance between particles, reducing interparticles’ hydrophobic/ hydrophilic attractions and van der Waals forces. However, the anomalous dispersion phenomenon of hydrophobic microparticles was only demonstrated on one specific example of using 2 μm-size polystyrene microsphere as core particle. Whether the anomalous dispersion phenomenon was extendable to other conditions or other hydrophobic microparticle systems was still unclear. The lacking of understanding limits the application scopes based on the nanospikes-mediated anomalous dispersion phenomenon. Therefore, systematic investigation on the anomalous dispersion phenomenon of hydrophobic microparticles including the effects of hydrophobic tethers, particle sizes, material compositions and core particle morphologies would extend the anomalous dispersion approach to a wider range of applications instead of relying on conventional surfacants.

**Figure 1:**
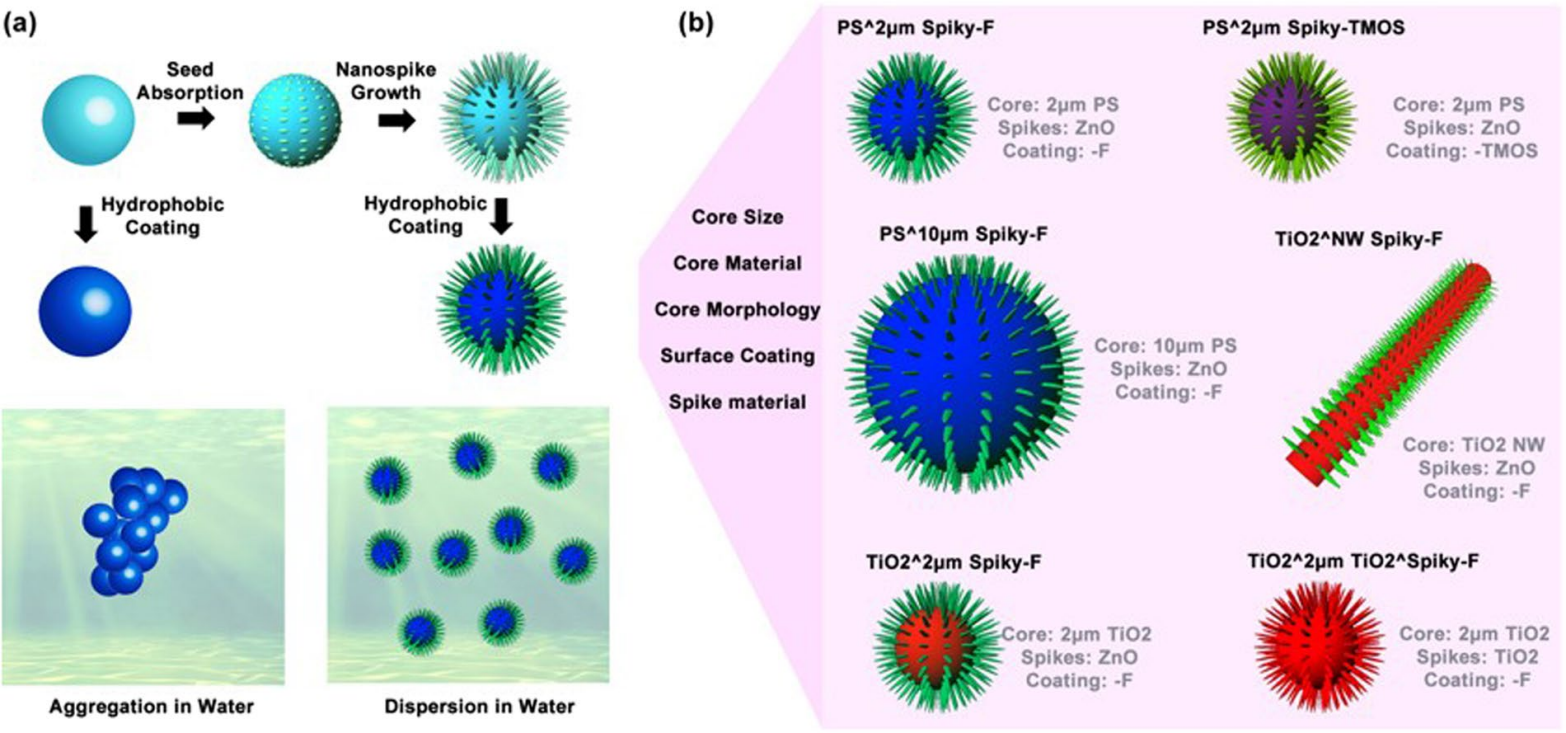
**(a)** Schematics showing the fabrication process of spiky micro-objects and process of hydrophobic coating. Typically, smooth micro-objects were firstly coated with ZnO nanoparticles and then nanospikes were grown on particle surface through hydrothermal approach. The smooth micro-objects and spiky micro-objects were coated with hydrophobic tethers, respectively, to produce hydrophobic micro-objects. The hydrophobic micro-objects without nanospikes tend to aggregate in water, while hydrophobic spiky micro-objects displayed anomalous dispersity in water. **(b)** Illustration of the investigation of the anomalous dispersion phenomenon on different particle systems with different hydrophobic coating, particle sizes, material compositions and core particle morphologies.

In this work, the anomalous dispersities of different hydrophobic spiky micro-objects including a broad range of conditions were systematically explored (Fig. 1), and the applications of hydrophobic spiky micro-objects for oil emulsion cleaning were demonstrated as example to show the advantage of nanospikes-mediated anomalous dispersities. Nanospikes were fabricated on the surface of micro-objects through hydrothermal approach, and then the particles were coated with hydrophobic tethers. The particle dispersities were characterized with photography, optical microscopy and scanning electron microscopy (SEM). The results show that the anomalous dispersion phenomenon was universally observed on hydrophobic spiky micro-objects with different hydrophobic coating, particle sizes, material compositions and core particle morphologies, while hydrophobic micro-objects without nanospikes aggregated severely in water. In addition, hydrophobic spiky micro-objects were applied for oil spills absorption and oil emulsion cleaning. Due to the presenting nanospikes, the spiky micro-objects displayed anomalous dispersity in water without compromising surface hydrophobicity, providing higher emulsion cleaning capability than the smooth micro-objects without nanospikes which aggregated in water with limited access to the oil emulsion. This work offers unique insight on the nanospikes-mediated anomalous dispersion phenomenon of hydrophobic micro-object and its applicability and application scopes of different hydrophobic particle systems without relying on traditional surfactants.

## Results and Discussions

**Figure 2:**
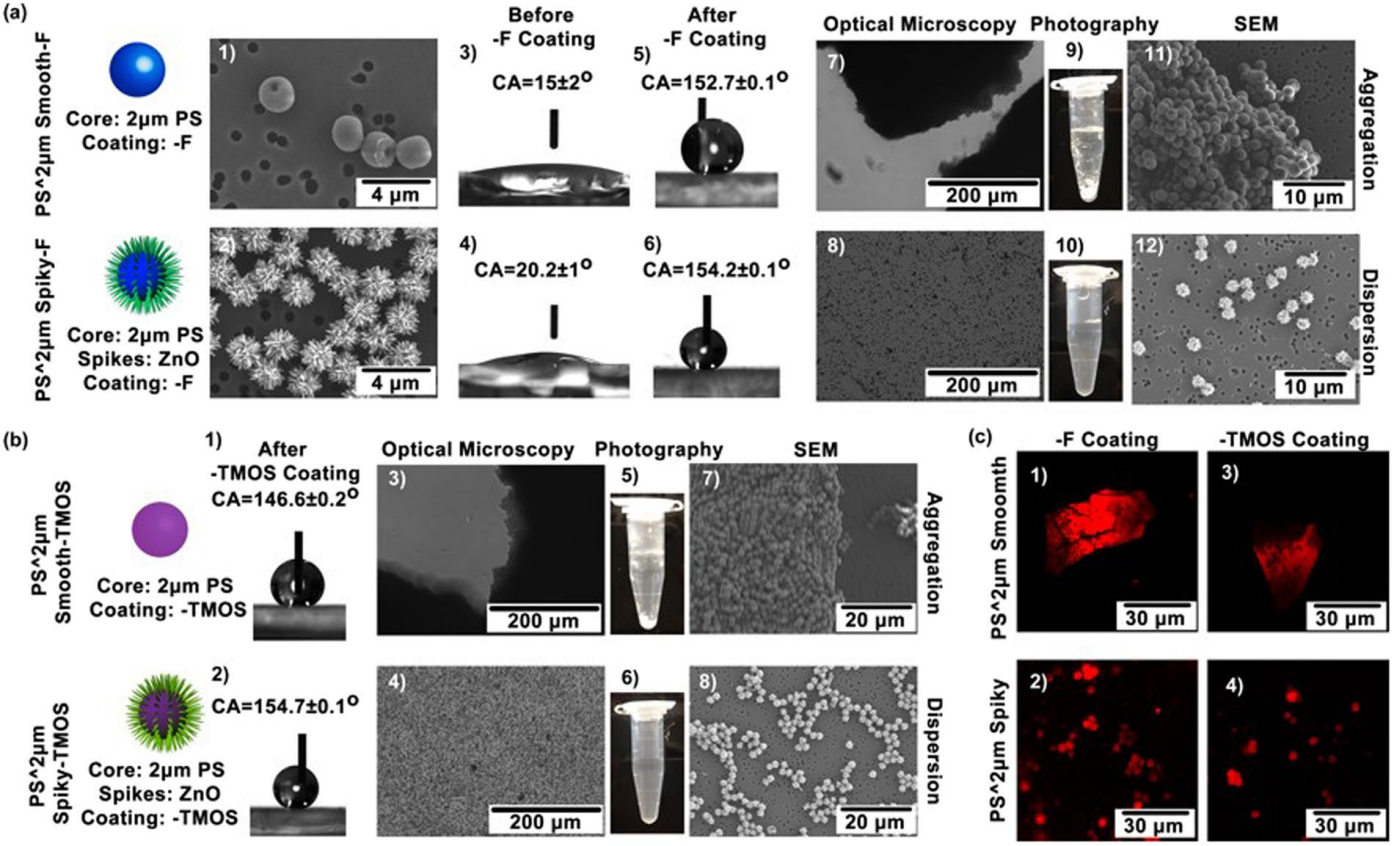
**(a)** Particles morphologies and dispersion profiles of PS^2 μm Smooth-F and PS^2 μm Spiky-F. (1–2) SEM images showing particle morphologies. (3–6) Water contact angle on particles film before and after PFES conjugation. (7–12) Photography, optical microscopy, and SEM imaging showing the particles’ dispersity profiles. **(b)** Particles’ dispersion profiles of PS^2 μm Smooth-TMOS and PS^2 μm Spiky- TMOS. (1) and (2) Water contact angle on particles film before and after TMOS conjugation. (3–8) Photography, optical microscopy, and SEM imaging showing the particles’ dispersity profiles. **(c)** Confocal fluorescence images showing the absorption of hydrophobic QDs (red fluorescence) to hydrophobic particles in water.

As the first example, polystyrene (PS) microbeads of 2 μm in diameter were functionalized with ZnO nanospikes and further coated to be superhydrophobic. The fabrication process was illustrated in Supporting Information S1, where smooth PS microspheres were initially decorated with ~35 nm ZnO nanoparticles which served as seed crystals for ZnO nanospike growth. In the second step, ZnO nanospikes were synthesized on the microsphere surface through hydrothermal route^23^. The growth of ZnO nanospikes was determined by the decorated ZnO nanoparticles but less dependent of the particle core. Therefore, coating different types of microparticles with ZnO nanospikes was feasible following similar fabrication process. The spiky particles were further conjugated with hydrophobic organic tethers such as 1H,1H,2H,2H-perfluorooctyltriethoxysilane (PFES). As control sample, smooth particles without nanospikes were functionalized to be hydrophobic using similar approach. The morphology of PFES-conjugated smooth particles (PS^2 μm Smooth-F) and spiky particles (PS^2 μm Spiky-F) were imaged with scanning electron microscopy (SEM) as shown in Fig. 2a1 and a2. The ZnO nanospikes protruding from the particle surface were revealed to be 20–40 nm in diameter and 400–800 nm in length. To confirm hydrophobicity after PFES conjugation, the wetting behavior of water on the microparticles-constructed thin film was investigated. The microparticles were deposited onto a glass substrate and allowed to dry. A drop of water was applied on top of the thin film surface and the static contact angle was measured. Before PFES coating, the water drop spread on both smooth particle film and spiky particle film with contact angle of 15 ± 2° and 20.2 ± 1°, respectively (Fig. 2a3 and a4). After PFES conjugation, the PS^2 μm Smooth-F film displayed contact angle of 152.7 ± 0.1°, and PS^2 μm Spiky-F exhibited contact angle of 154.2 ± 0.1°, indicating that the particles were successfully functionalized with PFES becoming superhydrophobic (Fig. 2a5 and a6). To examine the dispersity of these hydrophobic particles in aqueous solution, PS^2 μm Smooth-F and PS^2 μm Spiky-F were dried and re-suspended with DI water. Precipitation of PS^2 μm Smooth-F was readily observed at the bottom of the vial (Fig. 2a7), while PS^2 μm Spiky-F appeared to suspend forming uniform colloidal solution (Fig. 2a8). The particle dispersion profiles were revealed with optical microscopy by transferring the solution containing particles to a well plate. As shown in Fig 2a9, the PS^2 μm Smooth-F formed bulky aggregation in water, while PS^2 μm Spiky-F were dispersed with particles separating from each other (Fig. 2a10). In addition, the particles solutions were filtered by a nano-porous membrane to deposit the particles on substrate for SEM imaging. The filtration allowed immediate removal of water and hence preserved particle dispersion profiles. Similar to the results of optical imaging, aggregation of PS^2 μm Smooth-F was observed (Fig. 2a11), while PS^2 μm Spiky-F displayed dispersed profiles on the supporting membrane (Fig. 2a12). These results suggested that the hydrophobic microparticles functionalized with nanospikes exhibited anomalous dispersity in water, consisting with previous report.

This anomalous phenomenon has been hypothesized and theoretically simulated that the surface nanospikes remarkably prevent interparticle contact by the intercalation of nanospikes into the other particles’ interstitial spaces^24^. Accodring to a Cassie-Baxter wetting model, the attractive potentials between particles including interparticle van der Waals and hydrophilic/hydrophobic interactions became lower than the repulsive potential due to the steric hindrance of nanospikes. Therefore, aggregation was energetically unfavorable so that hydrohpobic spiky particles were effectively separated even in water.

To evaluate whether the anomalous dispersity is appliable to different hydrophobic tethers, the particles were conjugated with trimethoxy(7-octen-1-yl)silane (TMOS). Similarly, both smooth and spiky particles became superhydrophobic after conjugation, as the contact angle of water on PS^2 μm Smooth-TMOS film was 146.6 ± 0.2°, and 154.7 ± 0.1° on PS^2 μm Spiky-TMOS (Fig. 2b1 and b2). When PS^2 μm Smooth-TMOS was supplemented with water, these particles aggregated severely, and the results were verified with optical microscopy and SEM. Again, the PS^2 μm Spiky-TMOS readily dispersed in water, contrasted to the smooth particles (Fig. 2b3 and b8).

**Figure 3:**
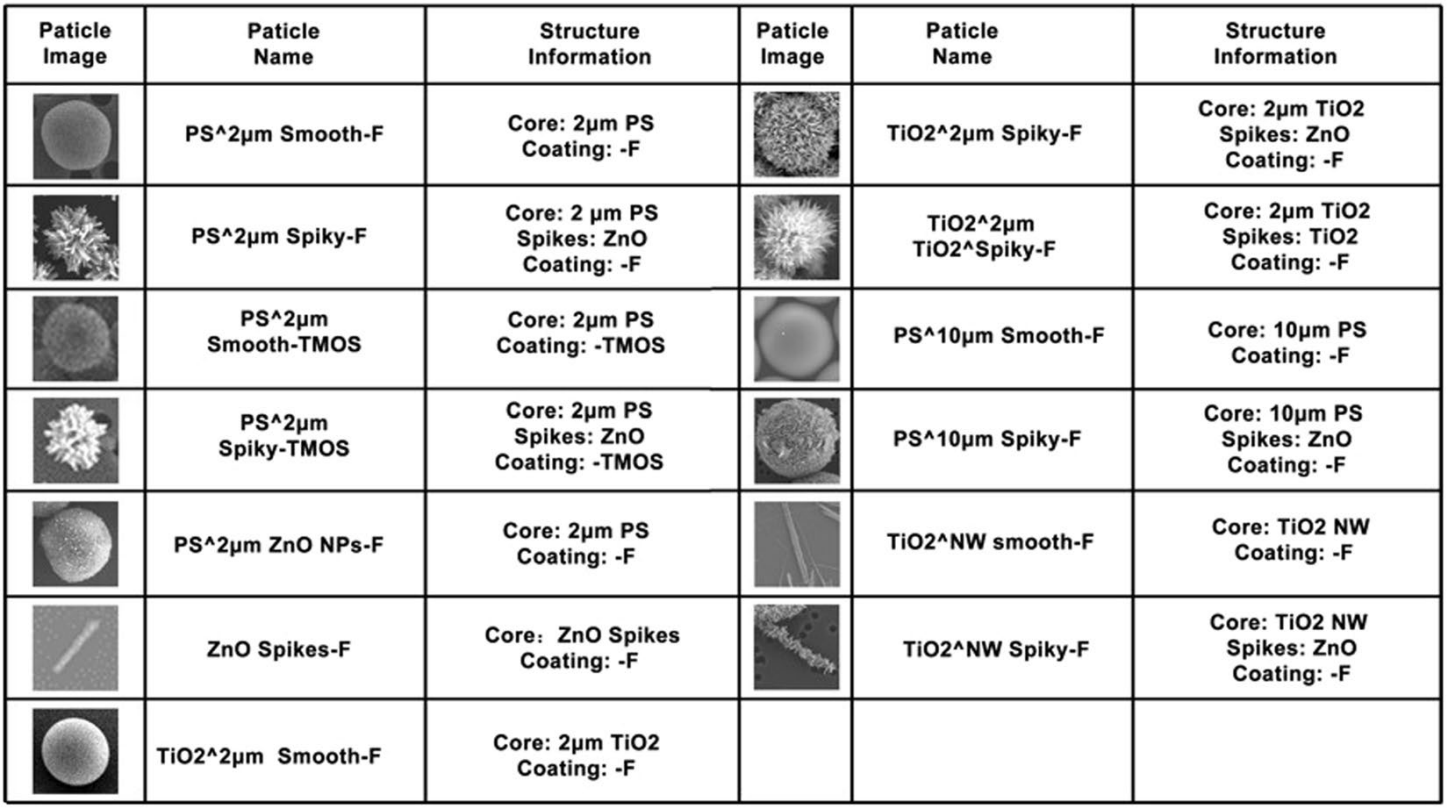
Information of different smooth and spiky particles.

To confirm the particles’ surfaces were hydrophobic after PFES or TMOS conjugation, hydrophobic quantum dots (QDs, red fluorescence) were employed as probes to test the absorption capability of hydrophobic substances by particle surfaces (Supporting Information S2). The hydrophobic QDs were injected to the aqueous solution of PS^2 μm Smooth-F, PS^2 μm Smooth-TMOS, PS^2 μm Spiky-F or PS^2 μm Spiky-TMOS. After mixing, all the particles exhibited red fluorescence as revealed via confocal fluorescence microscopy (Fig. 2c2–c4), indicating hydrophobicity of the particle surface. The particle images and structure information of different smooth and spiky particles were briefly summarized in Fig. 3.

**Figure 4:**
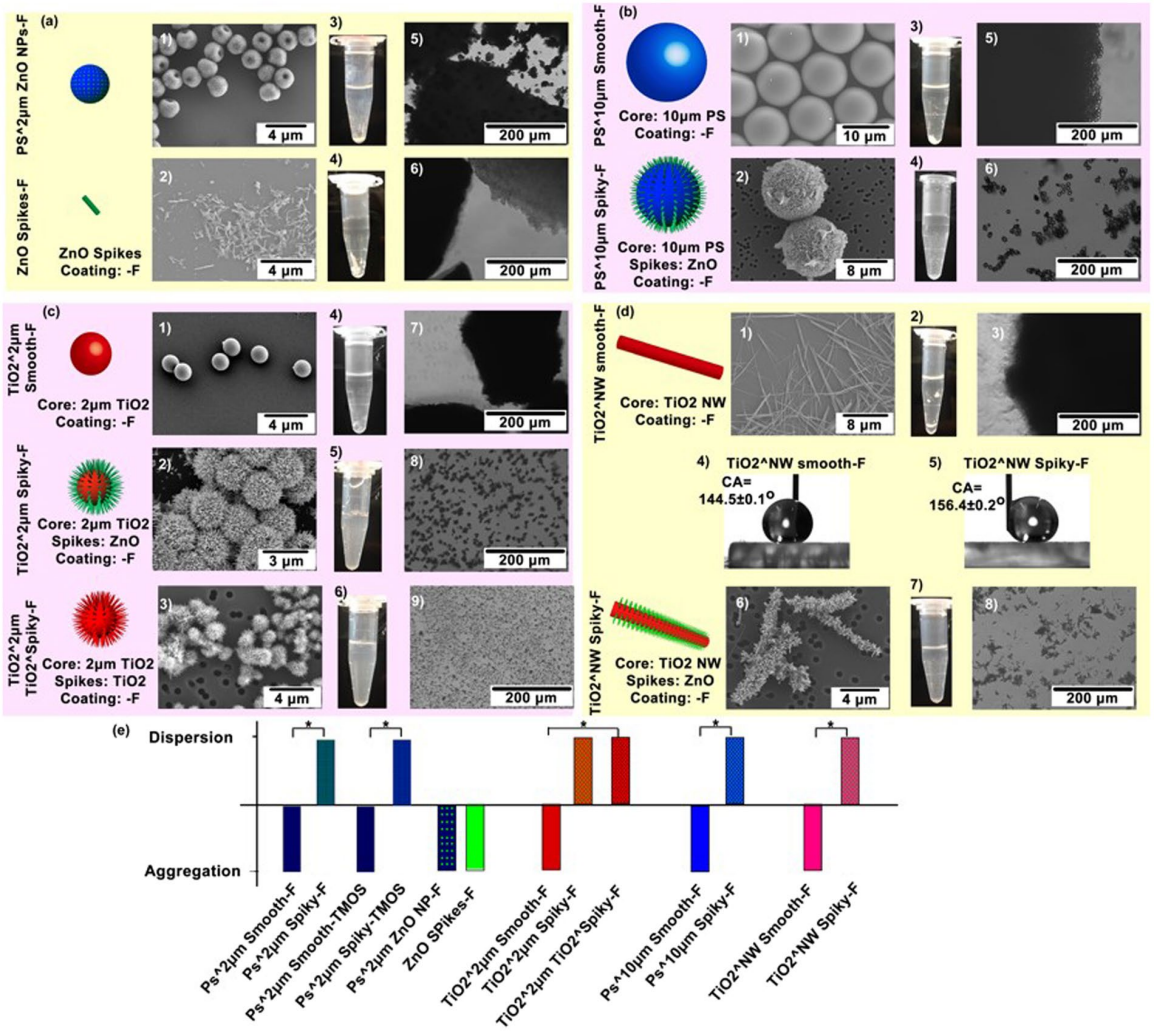
Water-dispersities of different types of hydrophobic micro-objects covered with or without nanospikes. **(a)** SEM images of PS^2 μm ZnO NPs-F and ZnO Spikes-F, and photographs and optical microscopic images showing their dispersities in water. PS^2 μm Spiky-F **(b)** SEM images of PS^10 μm Smooth-F and PS^10 μm Spiky-F, and photographs and optical microscopic images showing their dispersities in water. **(c)** SEM images of TiO_2_^2 μm Smooth-F, TiO_2_^2 μm Spiky-F and TiO_2_^2 μm TiO_2_^Spiky-F, and photographs and optical microscopic images showing their dispersities in water. **(d)** SEM images of TiO_2_^NW Smooth-F and TiO_2_•NW Spiky-F, and photographs and optical microscopic images showing their dispersities in water. Static contact angle measurement of water on TiO_2_^NW Smooth-F film and TiO_2_•NW Spiky-F film, confirming their hydrophobicities. **(e)** Diagram summarizing the dispersity/aggregation states in water of different hydrophobic particles with or without nanospikes.

To evaluate whether the anomalous dispersity was due to nanospikes, 2 μm smooth PS microspheres attached with nanoparticles (without nanospike growth, Fig. 4a) were conjugated with PFES to be hydrophobic and tested. These particles were hardly dispersed in water as revealed by optical microscopy. Similarly, ZnO nanospikes without attaching to microparticles (Fig. 4a) were conjugated with PFES, and they were found to be non-dispersible in water. These results indicating nanospike protruding from particle surface was the necessary geometry for anomalous dispersity. To explore whether anomalous dispersity of hydrophobic particles provided by surface nanospike functionalization was a universal phenomenon, microparticles with different sizes, material compositions or particle morphologies were tested. Firstly, PS microparticles of larger size (~10 μm in diameter) were functionalized with ZnO nanospikes followed by conjugation with PFES to produce hydrophobic spiky particles (PS^10 μm Spiky-F, Fig. 4b). These spiky particles readily dispersed in water as revealed via optical microscopy. Although precipitates were observed due to particle sinking, they could be easily re-suspended with gentle shaking. In contrast, 10-μm PS microparticles which were hydrophobically conjugated aggregated in water forming bulky precipitates.

After exploring the nanospikes-mediated anomalous dispersity on larger particles, whether this phenomenon was applicable to different core particle materials or different nanospike materials were investigated. TiO_2_ nano/micro-particles are one of the most promising materials that can be applied as photocatalysts^25,26^, drug carriers^27–29^, or components for cosmetics^30,31^, or sunscreens^32–34^. In this regard, 2 μm TiO_2_ microparticles were functionalized with ZnO nanospikes and then hydrophobically conjugated with PFES (TiO_2_^2 μm Spiky-F). In addition, spiky particles comprised of both TiO_2_ core particle and TiO_2_ nanospikes were produced through hydrothermal growth of urchin-like TiO_2_ microparticles, which were further conjugated with PFES to be hydrophobic (TiO_2_^2 μm TiO_2_^Spiky-F). Both TiO_2_^2 μm Spiky-F and TiO_2_^2 μm TiO_2_^Spiky-F exhibited anomalous dispersity in water in spite of the hydrophobic nature of particle surface (Fig. 4c). This is in siginficant contrast to 2 μm TiO_2_ smooth microparticles (hydrophobically functionalized, TiO_2_^2 μm Smooth-F) which suffered from severe aggregation in water (Fig. 4c). These results suggest the anomalous dispersion phenomenon on hydrophobic particles were not solely restricted to PS microparticles or ZnO nanospikes. The anomalous dispersity was applicable to different core materials or spike materials such as TiO_2_ and potentially more other materials.

Besides zero dimensional microparticles, whether the nanospikes-mediated anomalous dispersion was extendable to one dimensional nanomaterials was explored. Dispersion of hydrophobic nanowires (NWs) or nanorods in water have been traditionally achieved using surfactant additives^35–39^. Here hydrophobic TiO_2_ NWs with length of 10–15 μm were employed as an example to study the anomalous dispersion phenomenon. TiO_2_ NWs with (TiO_2_^NW Spiky-F) or without nanospikes (TiO_2_^NW Smooth-F) were conjugated with PFES, and their superhydrophobicity was verified by testing water wetting behaviors. The NWs were deposited on glass substrate forming continuous film, and contact angle measurement was conducted. The water contact angle was 144.5 ± 0.1° for TiO_2_^NW Smooth-F film suggesting hydrophobicity, and 156.4° ± 0.2° for TiO_2_^NW Spiky-F film which was slightly higher due to hierarchical micro-/nano-size features. When suspended in water, the TiO_2_^NW Smooth-F formed agglomeration due to the hydrophobic attraction between nanowires (Fig. 4d). On the other hand, TiO_2_ NWs camouflaged with ZnO nanospikes (TiO_2_^NW spiky) formed dispersion in water without precipitation (Fig. 4d), suggesting nanospikes decreased the particle attraction between NWs. The aggregation/dispersion states of particles with various hydrophobic coating, sizes, materials and morphologies presented in Figs 2 and 4 were summarized in Fig. 4e. Micro-objects functionalized with nanospikes all presented anomalous dispersities in water in spite of different material compositions, sizes, or morphologies. In significant contrast, smooth micro-objects without nanospikes formed severe aggregation in aqueous solution, indicating surface nanospikes protruding from particle surface were the major contribution to the anomalous dispersity.

Interparticle attractions such as the van der Waals and hydrophobic interactions among particles are related to particle sizes, material compostions and particle morphologies. The nanospikes on particle surface provided steric hindrance that prevented particle collision^24^, and this steric effect is significantly related to the nanospike structure. The intercalation of hydrophobic nanospikes into the interstitial spaces of other spiky particles was energetically unfavorable, and thus the repulsive potential overcame the attractive potential between particles leading to particle dispersion.

**Figure 5:**
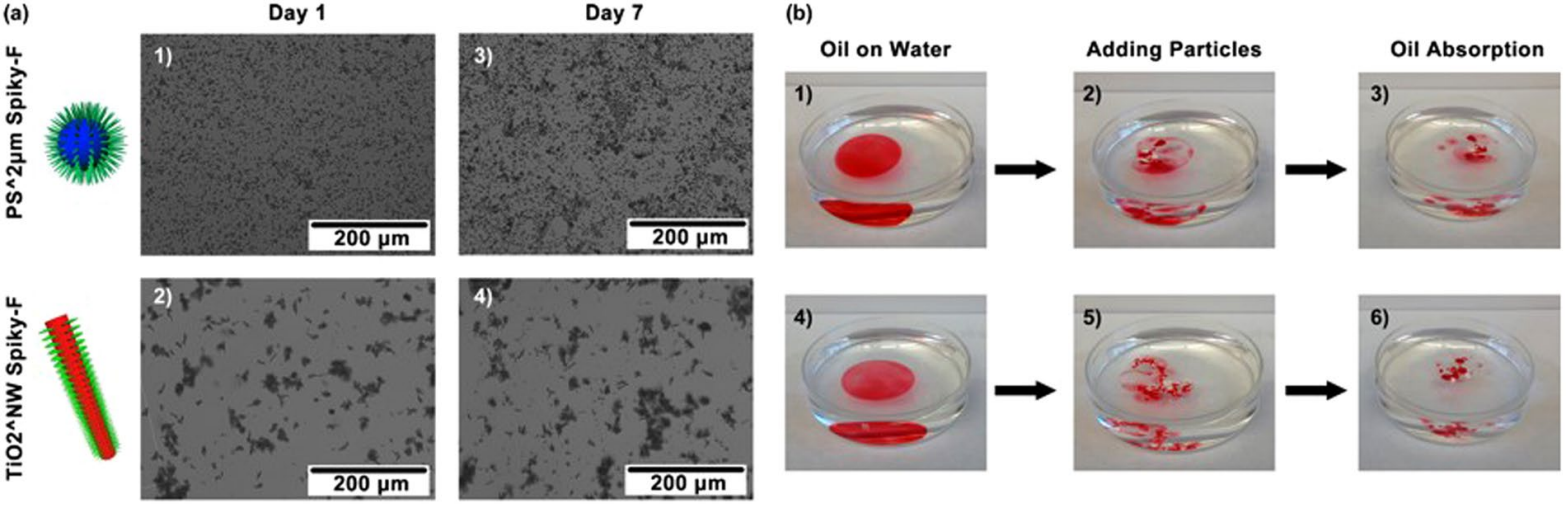
**(a)** Long-term dispersites (after one week) of PS^2 μm Spiky-F and TiO2^NW Spiky-F in water. **(b)** Absorption of oil spills (stained with red dye) floating on water using (1−3) PS^2 μm Spiky-F or (4−6) TiO2^NW Spiky-F. The absorption process was recorded.

The long-term stabilities of anomalous dispersity were investigated, where PS^2 μm spiky-F and TiO_2_^NW spiky-F were chosen as representatives of hydrophobic spiky micro-objects (Fig. 5a). The particle dispersions were stored for one week, and the particle dispersion profiles were examined with optical microscopy on both day one and day seven. It was observed that both PS^2 μm spiky-F and TiO_2_^NW spiky-F remained dispersed after one week, indicating the nanospike functionalization provided stable dispersion of hydrophobic micro-objects even with longer term.

To show the advantage of this surfactant-free hydrophobic particle dispersion approach, the capability of using spiky micro-objects to absorb oil spills were demonstrated with PS^2 μm spiky-F and TiO_2_^NW spiky-F as representatives. First, these hydrophobic spiky micro-objects were applied to absorb oil spills floating on water surface. Corn oil stained with Sudan red (for visualizing the oils) was employed as adsorbate to test the oil absorption capability of spiky particles. As shown in Fig. 5b, a drop of 60 mg corn oil was deposited on water surface. When 60 mg PS^2 μm Spiky-F or TiO_2_^NW Spiky-F were added on top of the oil film, the oils were gradually absorbed by the spiky micro-objects. The oil film continuously shrunk, and were completely absorbed by the hydrophobic micro-objects within 1 hour, demonstrating the oil absorption capability.

**Figure 6:**
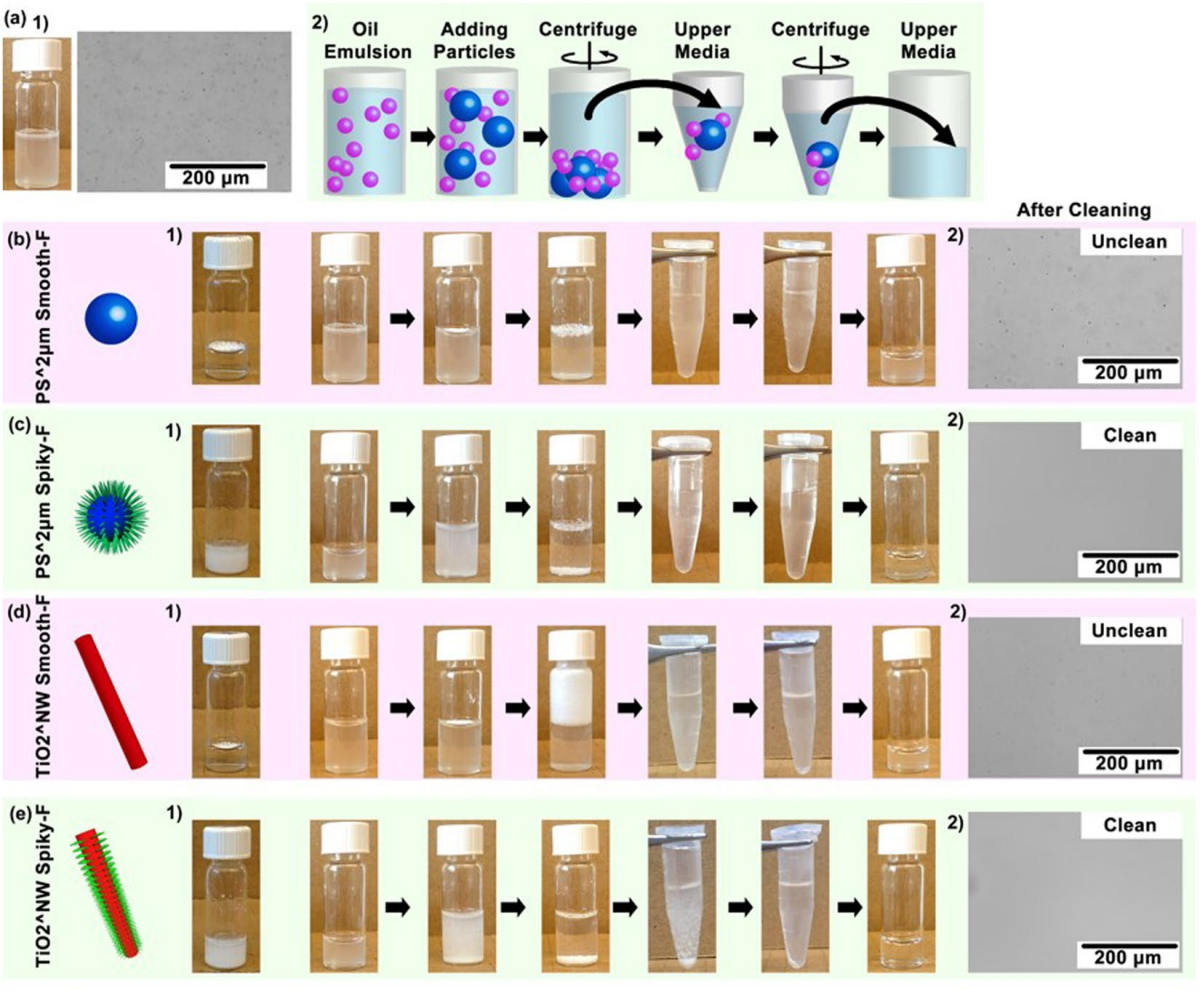
**(a1)** Photographic and optical microscopy images of 2% oil emulsion in water. **(a2)** Illustration of the process of oil emulsion removal using hydrophobic microparticles. **(b–e)** Photographic images showing the removal process of oil emulsion, and optical microscopic images showing the water cleaning results using **(b)** PS^2 μm Smooth-F, **(c)** PS^2 μm Spiky-F, **(d)** TiO2^NW Smooth-F and **(e)** TiO2^NW Spiky-F.

The hydrophobic spiky micro-objects were further explored for cleaning oil emulsion suspended in water. Oil-water mixture is challenging to clean *in situ* since it requires the hydrophobic oil absorbers to access to the oil emulsion in water, yet hydrophobic microparticles tend to aggregate in water with limiting exposed surface area. Here oil emulsion was produced by vigorously sonicating corn oil-water mixture to suspend oil drops homogeneously in water (Fig. 6a). The oil emulsion were stable and difficult to separate from the water even with high speed centrifugation at 14000 rpm for 5 min. The oil-water mixture solution became translucent, and the micro- or nano-drops of oil were observed to be smaller than 10 μm via optical microscopy. PS^2 μm Spiky-F and TiO_2_^NW Spiky-F were employed as representatives to clean 2 ml aqueous solution containing 2% oil emulsion, while PS^2 μm Smooth-F and TiO2^NW Smooth-F were used as controls to demonstrate the advantages of the presenting nanospikes (Supporting Information S3).

For hydrophobic spiky micro-objects, PS^2 μm Spiky-F (60 mg) or TiO2^NW Spiky-F (90 mg) were pre-suspended in 1 ml water. The pre-suspension of hydrophobic spiky micro-objects allowed increased contact between micro-objects and oil emulsions than directly adding dried micro-objects on water surface. To end up as 2 ml 2% emulsion solution, 1 ml aqueous solution of the pre-suspended spiky micro-objects were added into 1 ml 4% oil emulsion solution (Fig. 6c and e). For control micro-objects without nanospikes, dried powders of Smooth-F (60 mg) or TiO_2_^NW Smooth-F (90 mg) were directly supplemented to the emulsion solution since they were non-dispersible in water (Fig. 6b and d). The mixture solutions were vigorously vortexed for 5 min and allowed to stand for 30 min for oil emulsion absorption to the hydrophobic micro-objects. To separate the oil-microparticles complex from water, the mixture solutions were centrifuged at 5000 rpm for 10 min, and then the upper aqueous solution of each sample was withdrawn. Since the upper aqueous solutions might still contain a few oil-microparticles complex, the withdrawn upper solutions were treated with a second centrifugation at 14000 rpm for 5 minutes. After the second centrifugation, the upper solution of each sample was withdrawn again for examining water-cleaning profiles. The solutions treated with spiky particles, both Spiky-F and TiO_2_^NW Spiky-F, exhibited transparent and emulsion-free profiles as revealed via optical microscopy, indicating the oil emulsions were cleaned by the spiky micro-objects. In contrast, the solution treated with non-spiky particles, both Smooth-F and TiO_2_^NW Smooth-F, became less translucent yet still unclean. A large amount of emulsion drops were still readily observed in the withdrawn upper solutions, suggesting the solutions were not cleaned by the non-spiky control particles. The oil absorption results suggested the hydrophobic spiky particles provided higher emulsion-cleaning capabilities, which is due to the nanospikes offering the hydrophobic particles anomalous dispersities in water so that the surface of most individual particles was available for oil aborption. The hydrophobic particles without nanospikes aggregated severely in water with limited exposed surface for accessing to oil emulsions.

## Conclusion

To summary, the anomalous dispersity of hydrophobic spiky microparticles was attracting as surfactant-free dispersion strategy, yet whether this phenomenon was applicable to various hydrophobic microparticle systems was still unclear. Here the anomalous dispersities of different hydrophobic spiky micro-objects including a broad range of conditions such as different hydrophobic coating, particle sizes, material compositions and core particle morphologies were systematically investigated. The results indicate nanospikes consistently provided hydrophobic microparticles anomalous dispersity in various particle systems, suggesting the anomalous dispersion phenomenons was universally extendable various conditions. The hydrophobic spiky micro-objects were further explored for oil spills absorption and oil emulsion cleaning applications, and were found to provide higher emulsion cleaning capability compared to non-spiky particles. This is attributed to the increased dispersity of hydrophobic spiky micro-objects in water for accessing to the oil emulsion, while their surface hydrophobicity was preserved. Our work may offer better understanding on the nanospikes-mediated anomalous dispersion phenomenons and its extension to various hydrophobic microparticles systems. This approach may enable the generation of surfactant-free hydrophobic microparticles for a variety of applications such as emulsion adsorbers, chemical adsorbers, catalyst in chemical engineering and environmental science.

## Materials and Methods

### Fabrication of different hydrophobic spiky micro-objects

PS^2 μm ZnO NPs-F: 2 μm polystyrene microspheres (Polysciences, Inc.) with carboxyl group were supplemented in aqueous solution containing 0.2% ZnO nanoparticles (<35 nm, Sigma-Aldrich). The mixture solution was agitated at 400 rpm for 2 h. The ZnO nanoparticles were attached on the PS microsphere surface during the mixing and agitation. The unabsorbed ZnO nanoparticles were separated from the PS microspheres by centrifugation at 1000 rcf and washed with DI water for several times. The collected particles were treated with sonication for 2 hours to increase particle dispersity. This produced 2 μm PS microspheres attached with ZnO nanoparticles, and these particles were further conjugated with 1H,1H,2H,2H-perfluorooctyltriethoxysilane (PFES, Sigma-Aldrich) to produce PS^2 μm ZnO NPs-F. Briefly, the PS microspheres attached with ZnO nanoparticles were washed with anhydrous ethanol, and then suspended in anhydrous heptane/ethanol mixture solution (the volume ratio of anhydrous heptane: anhydrous ethanol was 5:1). The microparticles were then supplemented in anhydrous heptane/anhydrous ethanol mixture solution (the volume ratio of anhydrous heptane: anhydrous ethanol was 5:1) which contained 2% PFES. The mixture solution was stirred at 800 rpm at 80 °C for 12 hours. After reaction, the microparticles were washed several times with ethanol to remove unreacted PFES.

PS^2 μm Spiky-F: 2 μm PS microspheres attached with ZnO nanoparticles were supplemented with aqueous solution containing 25 mM zinc nitrate hydrate [Zn(NO_3_)_2_•6H_2_O, Sigma-Aldrich] and 25 mM hexamethylenetetramine (C_6_H_12_N_4_, HMTA, Sigma-Aldrich), and the mixture solution was stirred at 1000 rpm at 80 °C for 1 hour. This resulted in growth of ZnO nanospikes. The microparticles were then centrifuged and washed with DI water. The spiky microparticles were further conjugated with PFES to be hydrophobic following procedure described above.

ZnO Spikes-F: 2 μm PS microspheres attached with ZnO nanospikes were sonicated for 24 hours to remove the nanospikes from the microparticles. The nanospikes were separated from the microparticles via centrifugation and collected. They were further conjugated with PFES following procedure described above.

PS^2 μm Spiky-TMOS: The spiky microparticles were further conjugated with trimethoxy(7-octen-1-yl)silane (TMOS, Sigma-Aldrich). The spiky microparticles were washed with anhydrous ethanol, and then suspended in anhydrous heptane/ethanol mixture solution (the volume ratio of anhydrous heptane: anhydrous ethanol was 5:1). The microparticles were then supplemented in anhydrous heptane/anhydrous ethanol mixture solution (the volume ratio of anhydrous heptane: anhydrous ethanol was 5:1) which contained 2% TMOS. The mixture solution was stirred at 800 rpm at 80 °C for 12 hours. After reaction, the microparticles were washed several times with ethanol to remove unreacted TMOS.

PS^10 μm Spiky-F: 10 μm PS microspheres with carboxyl group (Polysciences, Inc.) were absorbed with ZnO nanoparticles, and ZnO nanospikes were grown on the particle surface following similar procedure as the 2 μm PS microspheres. The spiky microparticles were further conjugated with PFES to be hydrophobic following procedure described above.

TiO_2_^2 μm Spiky-F: TiO_2_ microparticles were deposited onto a supporting glass substrate and then coated with 20 nm ZnO layer via atomic layer deposition (ALD, Cambridge Nanotech). The particles were then collected, and ZnO nanospikes were fabricated on the particle surface, and were further conjugated with PFES following similar procedure as the 2 μm PS microspheres.

TiO_2_^2 μm TiO_2_^Spiky-F: 2 g TiO_2_ (Deguass P25) powders were mixed with 80 ml 5 M NaOH for 30 min, and then heated at 120 °C for 24 h in a teflon-lined stainless steel autoclave. The powder products were collected and dried at 60 °C in vacuum. For the second step, the powders were mixed with aqueous solution of 1 M NaOH (38 ml) and 30% H_2_O_2_ (25 ml, Sigma-Aldrich). The reaction was conducted at 150 °C for 8 h in a teflon-lined stainless steel autoclave. Powder products were collected and dried at 60 °C in vacuum again, and agitated with 0.05 M HNO_3_ solution. At the end, the products were washed and calcinated at 400 °C for 1 h.

TiO_2_^NW Spiky-F: TiO_2_ nanowires (Novarials) were absorbed with ZnO nanoparticles. ZnO nanospikes were fabricated on the particle surface, and were further conjugated with PFES following similar procedure as the 2 μm PS microspheres.

### Contact angle measurement on microparticle film

Microparticles (PS^2 μm Smooth-F, PS^2 μm Spiky-F, PS^2 μm Smooth-TMOS and PS^2 μm Spiky-TMOS) were deposited onto a glass substrate and dried at room temperature. The microparticles formed a thin film on the substrate. 5 μl drop of DI water was deposited on top of the microparticle thin film. Static Contact angle measurement was conducted with Goniometer measuring system to analyze the wetting properties of the microparticle thin film.

### Particle characterization

Particles were suspended in ethanol, and drop-casted onto a Si wafer substrate and allowed to dry. Samples were sputter-coated with Au-Pd, and imaged with SEM (Zeiss SUPRA 60).

### Characterization of particle dispersity in water

Hydrophobic microparticles were dried and placed in a 1.5 ml centrifuge tube, followed by supplementing DI water to the microparticles. The solution was shaken at 1500 rpm with vortex mixer (VWR) for 1 min. Particle dispersion was photographed. 200 μl particle solution was transferred to a 96-wells plate. Particle dispersion of typical area was imaged with optical microscopy (Mshot). To further examine particle dispersion and aggregation states, the particles were deposited onto track-etched polycarbonate membrane (Cyclepore) and the solution was immediately filtered away. This preserved particle dispersion profiles. The particles were collected on the porous membrane and dried for imaging with SEM.

### Long-term dispersity test

200 μl Aqueous solution containing particles (PS^2 μm Spiky-F or TiO_2_^NW Spiky-F) was placed in 96-wells plate. The particle dispersity was examined with optical microscopy on day one, and then stored without agitation for one week. On day seven, the particle dispersity profiles were imaged to examine the dispersion difference.

### Assay of hydrophobic QDs absorption to hydrophobic microparticles

Aqueous solution containing PS^2 μm Smooth-F, PS^2 μm Spiky-F, PS^2 μm Smooth-TMOS or PS^2 μm Spiky-TMOS was supplemented with drops of CdSe/ZnS core-shell type QDs aqueous solution (octadecylamine ligands functionalized, fluorescence λ_em_ 630 nm, 5 mg/ml in toluene, Sigma-Aldrich). The mixture solution was shaken vigorously with vortex mixer and was then transferred to a glass-bottom 96-wells plate. The particles were imaged with confocal fluorescence microscopy (Zeiss LSM).

### Application of hydrophobic microparticles for Absorbing oil spill on water surface

DI water was added to a petri dish, and 60 mg corn oil (Sigma-Aldrich) stained with sudan red was placed on top of the water surface. 60 mg PS^2um Spiky-F or TiO_2_^NW Spiky-F were slowly added to the oil surface. The oil drop was allowed to absorb to the microparticles for 1 hour. The oil absorption progress was recorded with photography.

### Application of hydrophobic microparticles for cleaning dispersed oil emulsion in water

Corn oil was mixed in DI water with the volume ratio of 2%, followed by sonication overnight to produce stable oil emulsion. Hydrophobic spiky particles (PS^2um Spiky-F and TiO2^NW Spiky-F) were pre-suspended in water, since they were dispersible in water. Hydrophobic smooth particles (PS^2um Smooth-F and TiO2^NW Smooth-F) were directly applied as dried powder since they aggregated in water. 1 ml Aqueous solution containing 60 mg PS^2um Spiky-F or 90 mg TiO2^NW Spiky-F were added into 1 ml 4% emulsion solution. This ended up to be totally 2 ml 2% emulsion solution. For hydrophobic smooth particles, 60 mg PS^2um Smooth-F or 90 mg TiO2^NW Smooth-F dried powders were directly added into 2 ml 2% emulsion solution. The mixture solutions were vigorously vortexed for 5 min and allowed to stand for 30 min for oil emulsion absorption to the hydrophobic micro-objects. To separate the oil-microparticles complex from water, the mixture solutions were centrifuged at 5000 rpm for 10 min, and then the upper aqueous solution of each sample was withdrawn. Since the upper aqueous solutions might still contain a few oil-microparticles complex, the withdrawn upper solutions were treated with a second centrifugation at 14000 rpm for 5 minutes. After the second centrifugation, the upper solution of each sample was withdrawn again for examining water-cleaning profiles with optical microscopy. The oil absorption progress was recorded with photography.

## Electronic supplementary material

Supporting Information
