# Supplementary material for: Nanospikes-mediated Anomalous Dispersities of Hydropobic Micro-objects and their Application for Oil Emulsion Cleaning
Source: Sci Rep. 2018 Aug 22;8:12600. doi: 10.1038/s41598-018-30339-3 (PMC6105594; doi:10.1038/s41598-018-30339-3)
Supplement: Supplementary file 1 — Supporting Information [file 41598_2018_30339_MOESM1_ESM.pdf]

Supporting Information for

Nanospikes-mediated Anomalous dispersities of Hydropobic Micro-objects and their Application for Oil Emulsion Cleaning

Hui-Jiuan Chen[1+], Chengduan Yang [1+], Tian Hang [1], Guishi Liu[1,2], Jiangming Wu[1], Di-an Lin[1], Aihua Zhang[1], Yan Li[1], Bo-ru Yang[1], Xi Xie[1,*]

[1] The First Affiliated Hospital of Sun Yat-Sen University; State Key Laboratory of Optoelectronic Materials and Technologies, School of Electronics and Information Technology; Guangdong Province Key Laboratory of Display Material and Technology, Sun Yat-Sen University, Guangzhou, China

[2]Department of Dermatology, Massachusetts General Hospital, Harvard Medical School, MA, USA

[+]These authors contributed equally to this work.

*To whom correspondence may be addressed. Corresponding to: Xi Xie, xiexi27@mail.sysu.edu.cn

S1. Fabrication process of different hydrophobic spiky microparticles

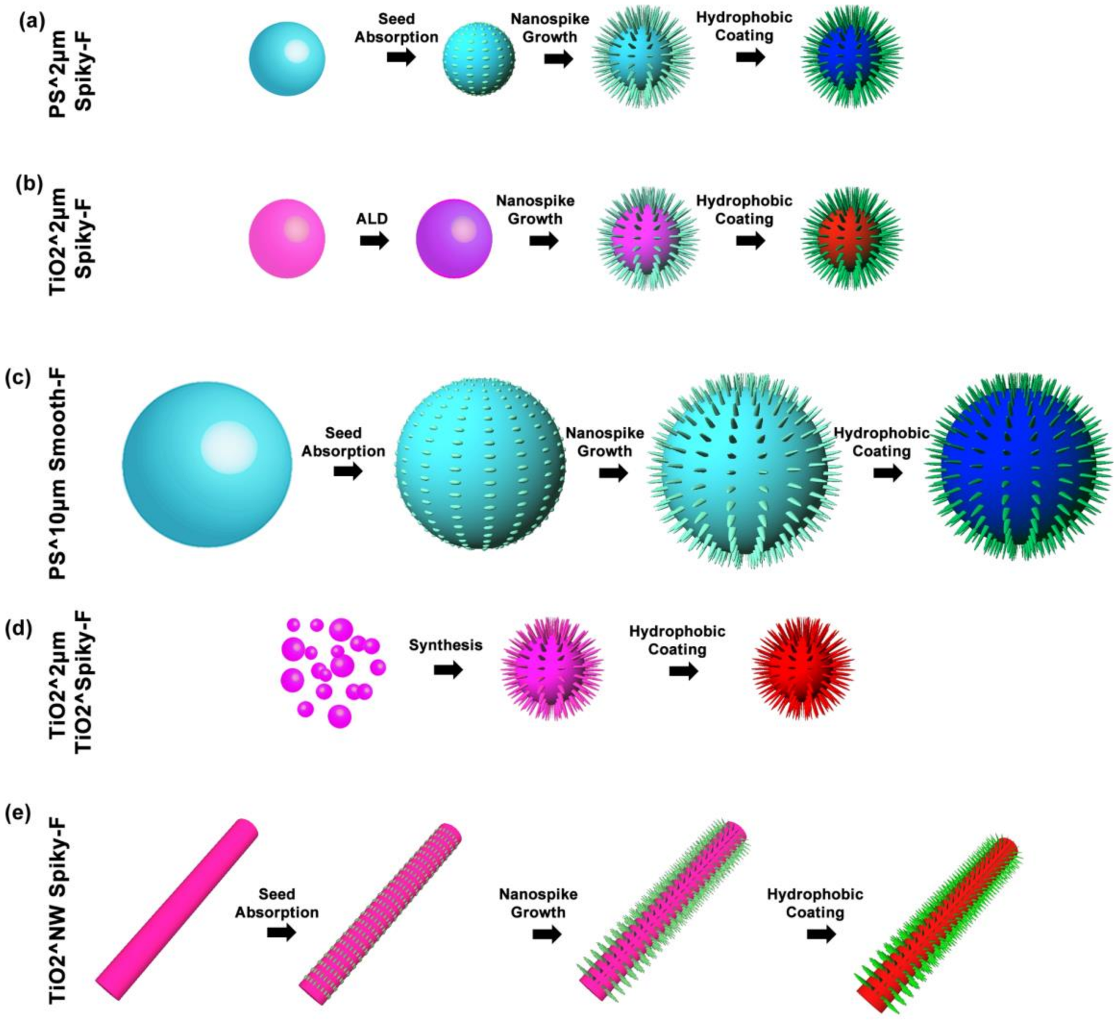

Figure S1.1. Illustration of the fabrication process of different hydrophobic spiky microparticles, including (a) using 2 µm PS microbeads as core particle and ZnO for nanospikes (PS^2um Spiky-F); (b) using 2 µm TiO_2 microspheres as core particle and ZnO for nanospikes (TiO2^2um Spiky-F); (c) using 10 µm PS microbeads as core particle and ZnO for nanospikes (PS^10um Spiky-F); (d) 2 µm TiO_2 core particles covered with TiO_2 nanpspikes through hydrothermal reaction (TiO2^2um TiO2^Spiky-F); (e) using 2 µm TiO_2 nanowire as core micro-object and ZnO for nanospikes (TiO2^NW Spiky-F).

S2. Supplemental information of hydrophobic QDs absorption assay.

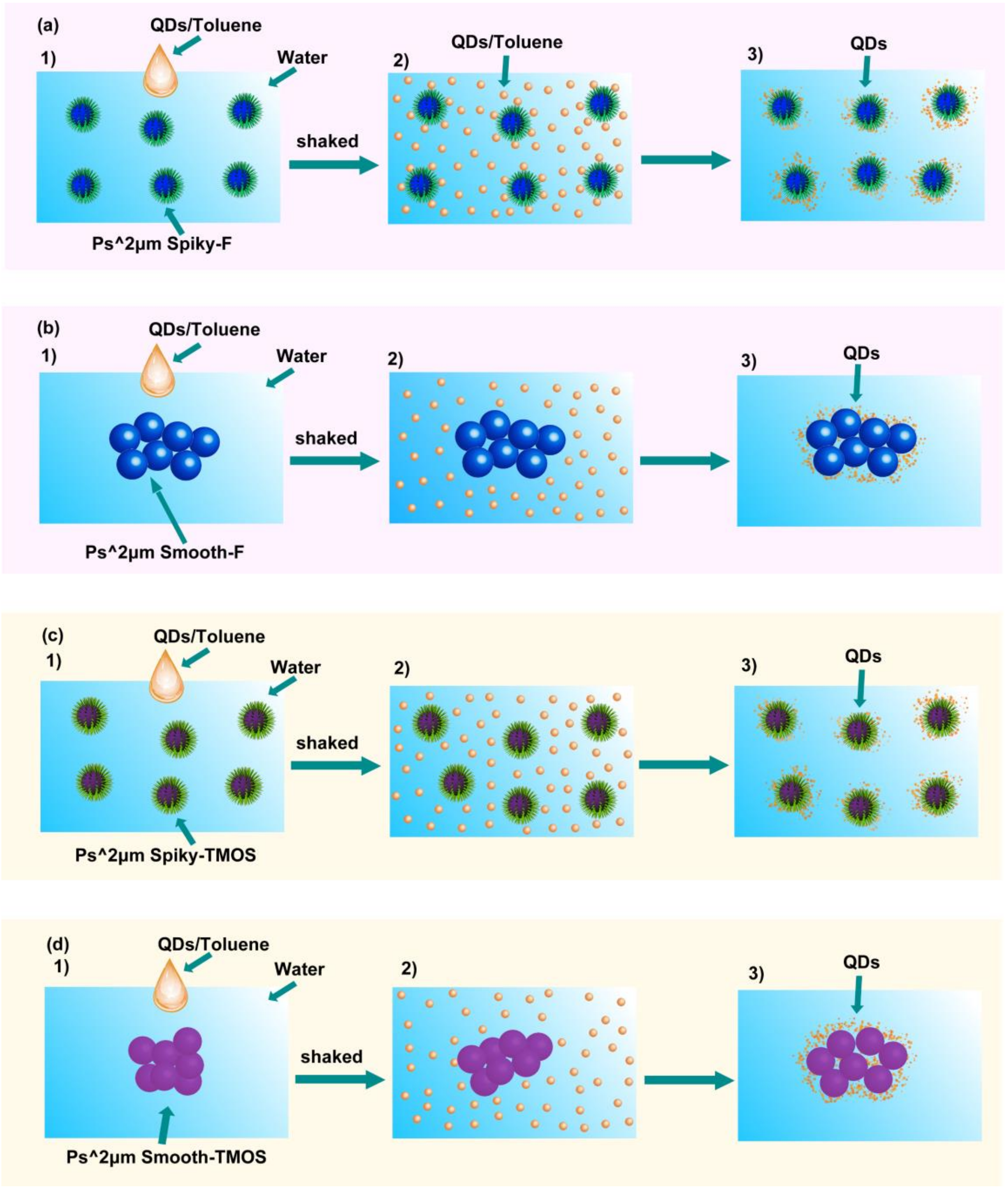

Figure S2.1. Illustration of QDs absorption assay of different hydrophobic 2 µm PS microparticles with or without spikes and different type hydrophobic coating, including (a) PS^2um Spiky-F; (b) PS^2um Smooth-F; (c) PS^2um Spiky-TMOS; (d) PS^2um Smooth-TMOS.

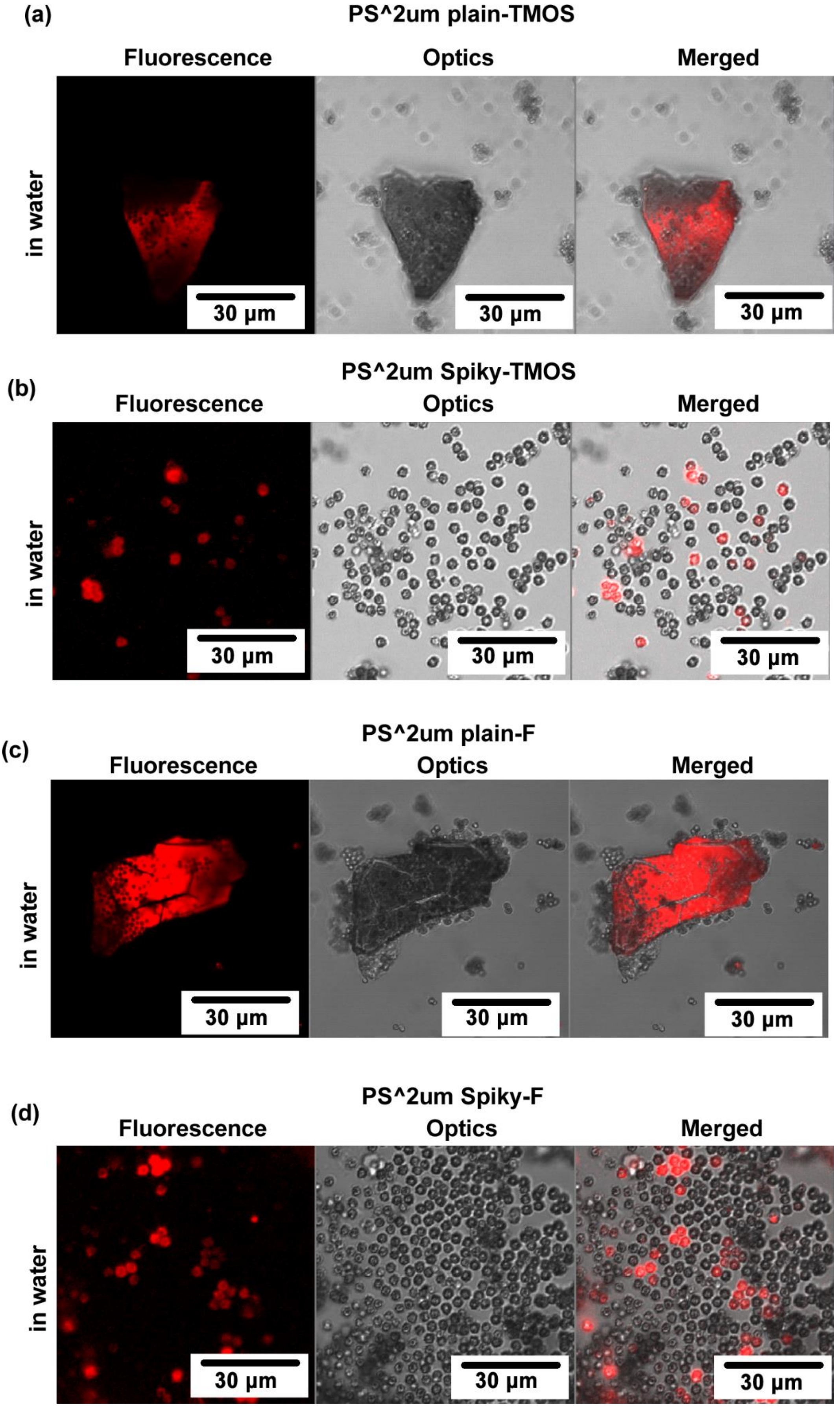

Figure S2.2. Confocal fluorescence images showing the absorption of hydrophobic

QDs to (a) PS^2um Plain-TMOS, (b) PS^2um Spiky-TMOS, (c) PS^2um Plain-F and (d) PS^2um Siky-F in water. Hydrophobic QDs attached on the particle surface presented red fluorescence signal, indicating particle surfaces were hydrophobic.

S3. Supplemental information of oil emulsions cleaning using hydrophobic microparticles.

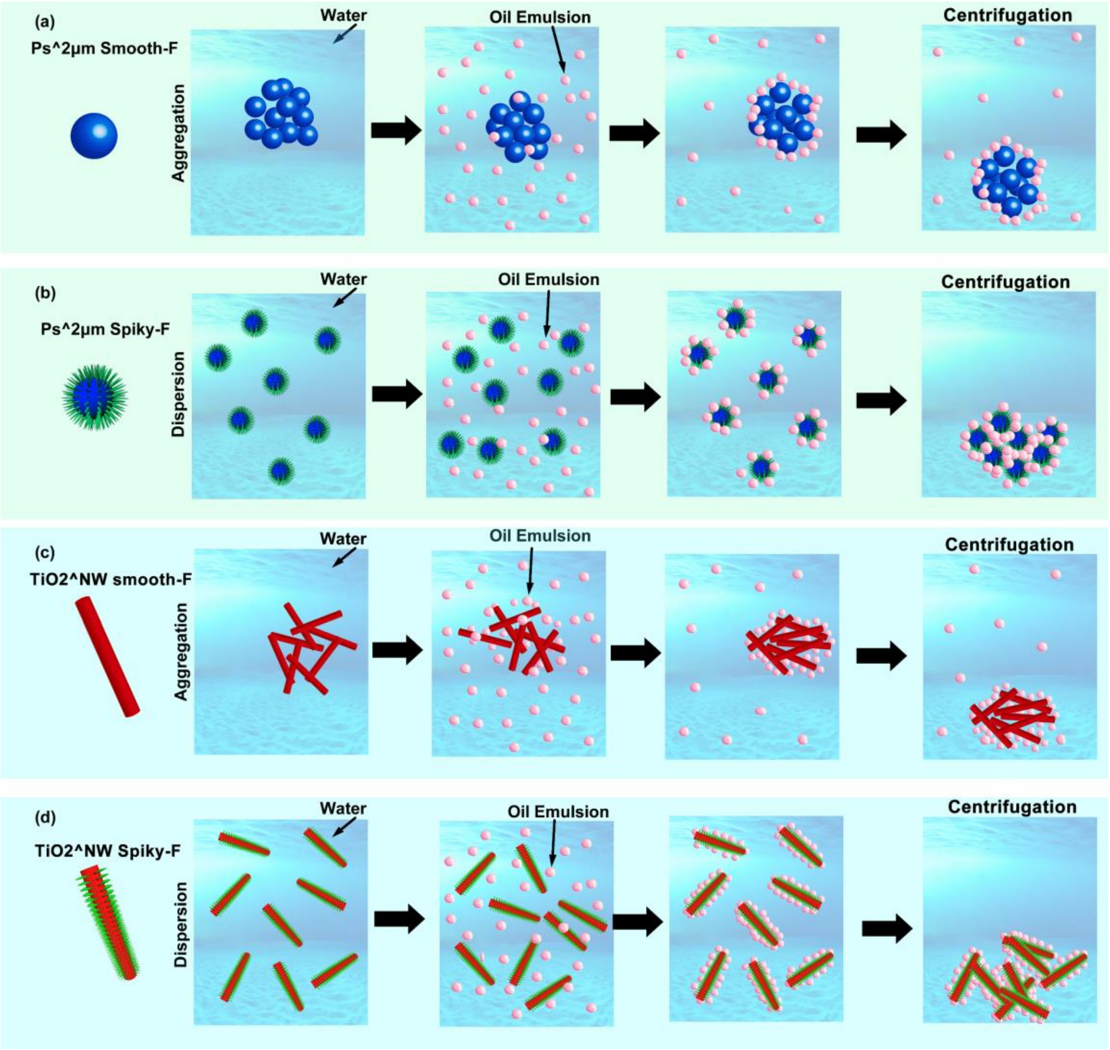

Figure S3. Illustration of oil emulsion cleaning in water by using (a) PS^2µm Smooth-F, (b) PS^2µm Spiky-F, (c) PS^2µm Spiky-F and (d) PS^2µm Smooth-F.

S4 **Supplemental information of the oil-wettability of PS^2 μm Spiky-F.**

In order to understand oil-wettability of spiky particles conjugated with PFES, the PS^2μm Spiky-F as an example was deposited onto a glass substrate and dried at room temperature. The PS^2μm Spiky-F formed a thin film on the substrate. 5 μl drop of corn oil was deposited on top of the PS^2μm Spiky-F thin film. Static Contact angle measurement presented the oil contact angle values of PS^2μm Spiky-F was about 0 degree meanwhile the oil contact angle values of PS^2μm Spiky was about 22.65° ±1.65° . The results demonstrated the PS^2μm Spiky-F present superoleophilicity.

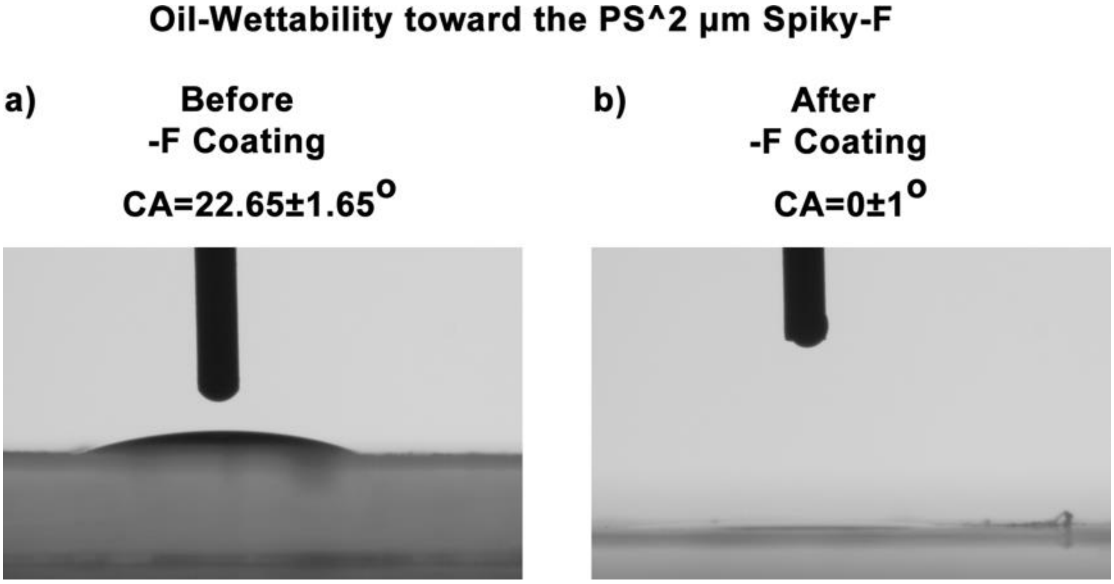

Figure S4 The oil-wettability of PS^2 μm Spiky microparticles **(a)** PS^2μm Spiky without fluorination. (b) PS^2μm Spiky conjugated with PFES.
